# Insights of the Population Structure and Breeding Biology of a Xanthid Crab *Etisus laevimanus* Randall, 1840, on the Rocky Intertidal Region of the Gujarat Coast

**DOI:** 10.1002/ece3.70605

**Published:** 2024-11-28

**Authors:** Bhavesh R. Shrimali, Krupal J. Patel, Ashish Patel, Dipak Kumar Sahoo, Mansour Shrahili, Mohammad Javed Ansari, Jigneshkumar N. Trivedi

**Affiliations:** ^1^ Department of Life Sciences Hemchandracharya North Gujarat University Patan Gujarat India; ^2^ Department of Veterinary Clinical Sciences College of Veterinary Medicine, Iowa State University Ames Iowa USA; ^3^ Department of Statistics and Operations Research, College of Science King Saud University Riyadh Saudi Arabia; ^4^ Department of Botany Hindu College Moradabad (Mahatma Jyotiba Phule Rohilkhand University Bareilly) Bareilly Uttar Pradesh India

**Keywords:** Arabian sea, Decapoda, population ecology, Saurashtra coast, Shivrajpur, Xanthidae

## Abstract

The population structure and breeding biology of a Xanthid crab, *Etisus laevimanus* Randall, 1840, a commonly occurring species on the rocky intertidal region of Saurashtra coast of Gujarat State, India, were investigated. Samples were collected from the intertidal region for 12 consecutive months during low tide using catch per unit effort in a 500 m^2^ area. The individuals were categorized into male, non‐ovigerous female, or ovigerous female. For fecundity estimation, the total number of eggs, size of eggs, and weight of egg mass were measured along with the carapace width and body weight of the respective ovigerous females. Sexual dimorphism was evident in the collected samples, with males being significantly larger than females. The overall as well as monthly sex ratio was female‐biased. Size frequency distribution showed a bimodal frequency distribution in males while being unimodal in females. The occurrence of ovigerous females throughout the year suggests that the species has a continuous breeding pattern with peaks from December to April. The total number of eggs, size of eggs, and weight of the egg mass showed a significantly positive correlation with the carapace width of the ovigerous females.

## Graphical abstract

The population structure and breeding biology of a Xanthid crab *Etisus laevimanus* Randall, 1840, were investigated; this is commonly occurring species on the rocky intertidal region of Saurashtra coast of Gujarat State, India. Size frequency distribution showed a bimodal frequency distribution in males while being unimodal in females. The occurrence of ovigerous females throughout the year suggests that the species has a continuous breeding pattern with peaks from December to April. The total number of eggs, size of eggs, and weight of the egg mass showed a significantly positive correlation with the carapace width of the ovigerous females.

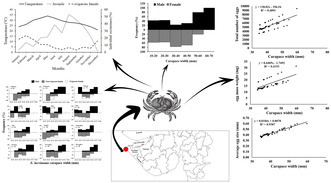

## 1 Introduction

Studies on populations generally address questions related to seasonal abundance, size, structure, sex ratio, and breeding periods that can be compared with other populations of the same species, genus, or other taxonomic level (Patel, Patel, Ali, et al. 2024; Patel, Patel, Gosavi, et al. 2024). Such comparisons are an important strategy to verify differences among populations and to understand the biological constraints shaping the structure of these populations (Branco et al. 2002). Seasonal variation in population, size distribution, density, abundance, frequency, sex ratio, natality, and mortality rates are prominent aspects to investigate population structure and breeding biology of marine crabs (Masunari and Dissenha 2005; Bezerra and Matthews‐Cascon 2007). Population ecology is the study of populations in relation to the environment, including environmental influences on population density and distribution, age structure, and population size. Moreover, the study of dominant populations may be very important to elucidate the structure, function of communities, and ecological stability of the species in its habitat, while adding to our knowledge of the species' biology (Litulo 2005; Takween and Qureshi 2005). Such studies in India have been started very recently (Patel, Vachhrajani, and Trivedi 2023; Patel, Patel, Ali, et al. 2024; Patel, Patel, Gosavi, et al. 2024), and the knowledge on the ecological aspects of marine crabs is scarce.

A total of six species of genus *Etisus* have been recorded, out of which, *E. laevimanus* Randall 1840, is the most commonly occurring species on the coastal region of Gujarat. The species is mainly found inhabiting live and dead corals and crevices of rocks or under the stones in the intertidal region. The morphological identification characteristics of the species are as follows: Carapace feebly convex, broader than long, and nearly glabrous; frontal margins feebly sinuous, slightly exceeding supraorbital angles; anterolateral margins with four smooth teeth behind exorbital angles; chelipeds slightly unequal, especially in male; minor cheliped similar to major cheliped, except for shorter palm; dactylus of cheliped spoon‐shaped; G1 thin, elongate; and tip strongly curved, rounded, and serrated, with subdistal short setae (Amer, Naruse, and Reimer 2022).

The presence of *E. laevimanus* is associated with increased species richness in its habitat, demonstrating its role as a keystone species in marine ecosystems (Goulletquer et al. 2014). The ecological role of this marine crab species is crucial for sustaining biodiversity within marine ecosystems. As an ecosystem engineer, it affects the habitat structure and resource availability, which supports various marine organisms. *Etisus laevimanus* enhances habitat complexity by burrowing and creating microhabitats, thereby contributing to the physical structure of its environment (Cribb et al. 2024). Such modification can increase environmental heterogeneity, enabling diverse species to flourish, thus promoting biodiversity. The species also plays a vital role in nutrient cycling by facilitating the breakdown of organic matter, which is crucial for maintaining ecosystem health by benefiting both primary producers and higher trophic levels (Cribb et al. 2024). By supporting diverse marine life, it contributes to the overall stability of marine ecosystems, which are increasingly threatened by human activities (Vinayaka et al. 2024).

While *E. laevimanus* plays a vital role in enhancing marine biodiversity, the ongoing threats from overfishing and habitat degradation pose significant risks to its populations and the ecosystems they support (Perez and Mendoza 1998). *Etisus laevimanus* is commonly found on the intertidal region of Shivrajpur on Gujarat coast, which is designated as a blue flag beach. However, the aspects of its population structure and breeding biology are yet unknown. Understanding crab population dynamics, reproductive strategies, and distribution factors might aid in evaluating their vulnerability to the effects of climate change, including increasing sea temperatures and alterations in habitat availability. Hence, the present study is focused on understanding the population structure and fecundity of *E. laevimanus* with the aim of understanding the population structure and breeding biology of *E. laevimanus,* which is the commonly occurring xanthid crab species on Saurashtra coast of Gujarat state, India. The study will fill the gap in the understanding of the ecology of some commonly occurring crabs on Gujarat coast, while providing a baseline data important in understanding the effects of continuously changing environment and habitat along with increasing anthropogenic pressure.

## 2 Materials and Methods

### 2.1 Study Area

**FIGURE 1:**
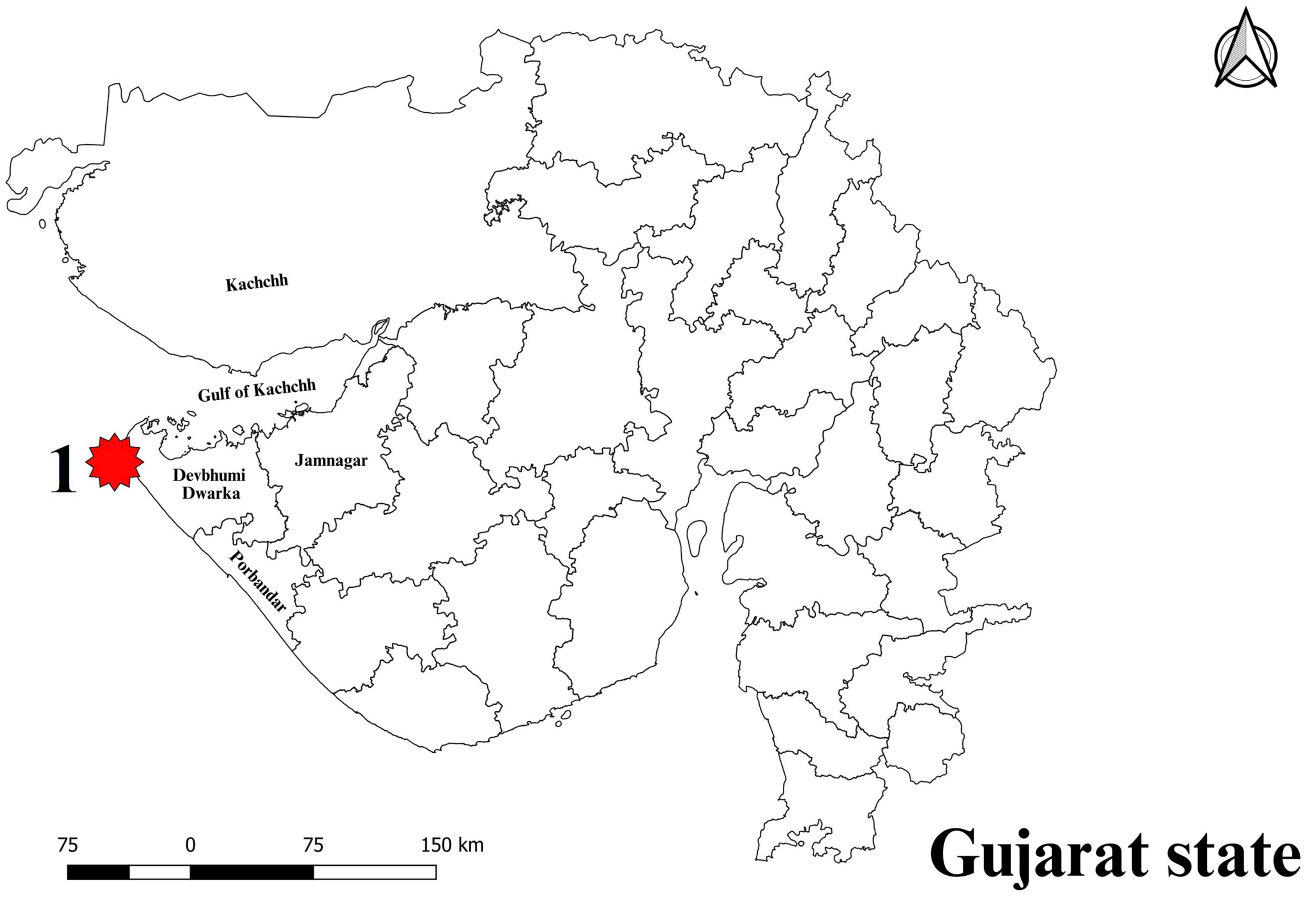
Map of the study area: Shivrajpur, Saurashtra coast, Gujarat, India.

Sample collection for the current investigation was conducted on the rocky intertidal region of Shivrajpur (22°12′21.97″ N 68°58′31.75″ E) of Devbhumi Dwarka district located at Saurashtra coast, Gujarat coast, India (Figure 1).

### 2.2 Field Methodology

Field work was conducted for 12 consecutive months (March 2019 to February 2020) during low tide time, and the month‐wise data was compiled into different seasons, viz., winter season (November to February months), summer season (March to June months), and monsoon seasons (July to October months), following Rao and Rama‐Sharma (1990). When the water receded, a 500m^2^ area was marked randomly on the intertidal region with the help of measuring tape and thoroughly scanned for the presence of *E. laevimanus* . The specimens occurring in this area were collected by catch‐per‐unit effort using a hand picking method for the time period of 4 h. Small rocks in the marked area were also upturned for the presence of the individuals, which is preferred by the species for shelter. Whenever an individual of *E. laevimanus* was encountered, it was collected and preserved in 10% formalin for further analysis in laboratory.

### 2.3 Laboratory Analysis

Firstly, the collected specimens were identified upto species level using the identification key provided by Amer, Naruse, and Reimer (2022). Further, the identified individuals were categorized into male, non‐ovigerous female, or ovigerous female (female individuals bearing eggs). The morphological characteristics were recorded in terms of carapace width (CW) and body weight (BW) using a digital vernier callipers (0.01 mm accuracy; Mitutoyo Digimatic Vernier Caliper 500‐196‐20) and a digital weighing scale (0.001 g accuracy; Accuris Mini weighing scale), respectively. Individuals that were smaller than the smallest ovigerous female from the collected samples were considered as juvenile individuals (< 34.22 mm), as already established (Mantelatto and Garcia 2001; Baeza et al. 2013).

For fecundity analysis, the methodology was adopted from Patel, Vachhrajani, and Trivedi (2023). Fecundity estimation was conducted by carefully removing the entire egg mass from the pleopods of the ovigerous female (*n* = 47) and putting in saline water (20 mL) for the following parameters: size of eggs (diameter), weight of egg mass, and total number of eggs.

The ovigerous females were initially weighed, along with the egg mass. The egg mass was subsequently removed and reweighed, with the difference recorded as the weight of the egg mass. The total number of eggs was determined by carefully collecting the egg mass from the pleopods of ovigerous females using a brush and forceps, transferring it to 20 mL of seawater, and gently mixing to prevent breakage and ensure even distribution. To find out the total number of eggs in the already mentioned solution, three samples of 2 mL each were collected in a petri dish and examined under a stereomicroscope. The mean of the total egg count from the three samples was multiplied by the dilution factor (DF = 10) (Litulo 2005) to estimate the total number of eggs. The egg diameter (*n* = 10) of each ovigerous female was determined with the help of an ocular micrometre mounted to a stereo microscope (Saher and Qureshi 2010).

### 2.4 Data Analysis

The species were first grouped in 10 mm size class intervals starting from 10.00 mm to 70.00 mm CW for the purpose of understanding overall size frequency distribution. Further, to check whether the data is normally distributed or not, a Shapiro–Wilk test was conducted, which suggested that the data distribution was not normal (*p* < 0.01). Following the non‐normal distribution of the data, non‐parametric tests were conducted. The Kruskal–Wallis (KW) test was performed to compare the mean values of the carapace width between male, non‐ovigerous female, and ovigerous females. When a significant difference was obtained (*p* < 0.05), Dunn's post hoc test (a multiple comparison analysis) was conducted. To understand the monthly variation in the size (CW) and sex composition of *E. laevimanus* , data on individuals' carapace width and sex was plotted. Chi‐square test (*χ* ^2^) was performed to find out the difference in the ratio of male and female individuals (ovigerous and non‐ovigerous females). To find out the effect of temperature on the breeding and juvenile settling of *E. laevimanus* , monthly data of the incidence of juvenile and ovigerous females against ambient temperature was plotted. A Pearson's correlation analysis was performed to examine the relationship between the mean ambient temperature and the relative juvenile frequency.

The relationship between the morphology of eggs and the crabs was investigated by performing regression analysis. At a significance level of *p* < 0.05, the statistical results were considered to be statistically significant. For all the statistical analysis, MS Excel and PAST software (version 4.03) were used.

## 3 Results

**FIGURE 2:**
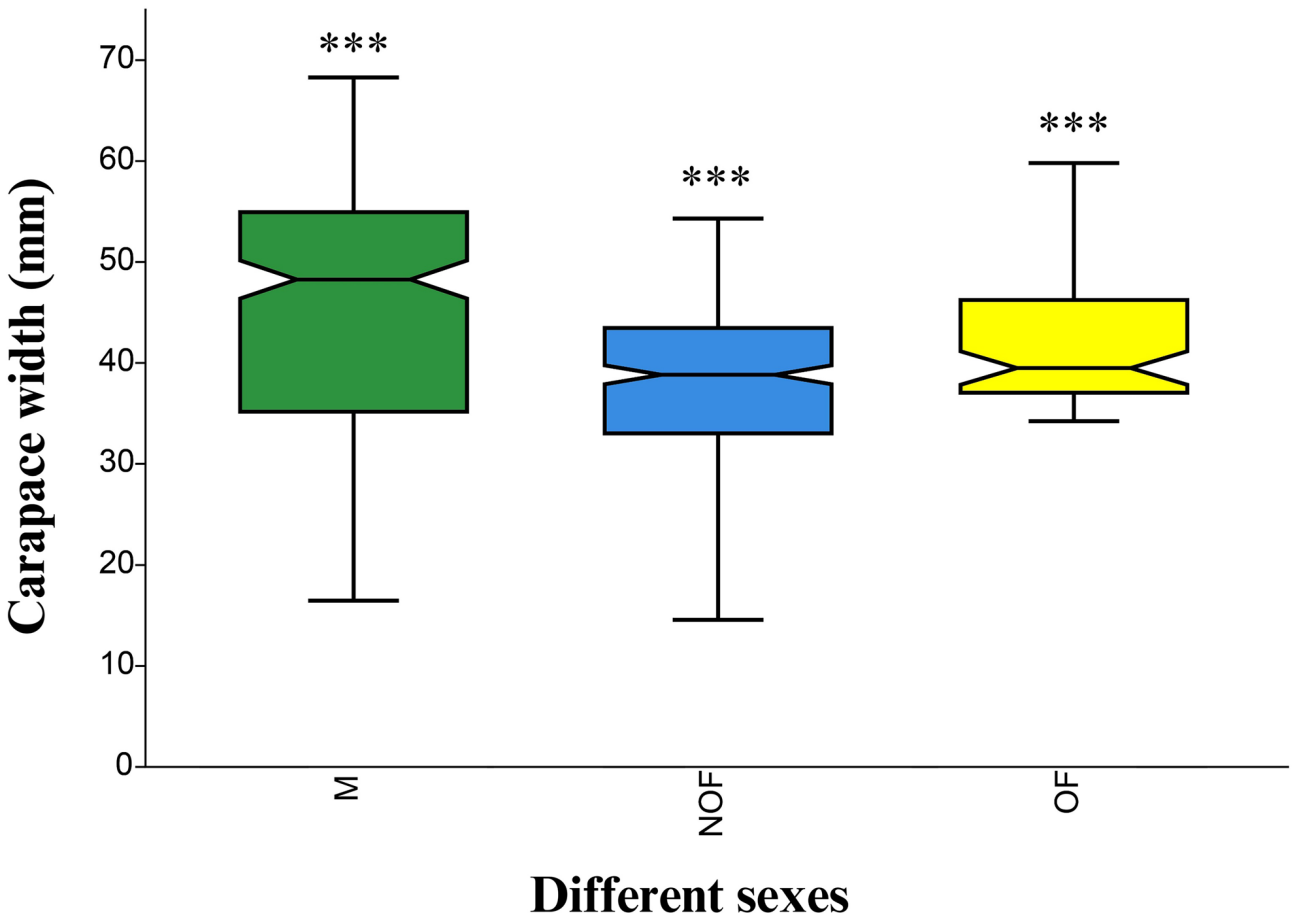
Carapace width of individuals from different sexes of *Etisus laevimanus* from Shivrajpur, Saurashtra coast, Gujarat, India. (Box = 25th and 75th percentiles, midline = mean, whiskers = minimum and maximum values; ***Significant level if *p* < 0.001).

During the study, a total of 655 individuals were sampled, out of which 273 were male (41.68%), 306 were non‐ovigerous female (46.72%), and 76 were ovigerous females (11.60%). The CW of male individuals ranged from 16.67 to 68.28 mm CW, while in the case of female individuals, the CW of non‐ovigerous females and ovigerous females ranged from 14.57 to 54.3 mm and 34.22 to 59.8 mm, respectively. A prominent sexual dimorphism was observed with the mean size of male individuals being significantly larger as compared to female individuals (non‐ovigerous females and ovigerous females) (Kruskal–Wallis, *H* = 78.07, *p* < 0.001). Further, Dunn's post hoc test revealed that the mean size of ovigerous female was also significantly larger as compared to non‐ovigerous female individuals (Bonferroni‐corrected, *p* < 0.001) (Figure 2).

The findings of the current study revealed that the overall sex ratio (1:1.43) was female‐biased, which differed significantly from the expected 1:1 proportion (*χ* ^2^ = 78.07, *p* < 0.001) (Table 1). The monthly sex ratio was also female‐biased except in the October month. Occurrence of ovigerous females was recorded in all the months of the year, suggesting a continuous pattern of breeding with the peak from December to April (Table 1).

**TABLE 1:** Total number of *Etisus laevimanus* specimens collected from Shivrajpur, Saurashtra coast, Gujarat state, India.

Month	M	%	NOF	%	OF	%	Total	M:F
January	20	36.86	24	44.24	10	18.90	54	1:1.71
February	25	49.02	14	27.45	12	23.53	51	1:1.04
March	33	45.21	27	36.99	13	17.81	73	1:1.21
April	23	42.59	22	40.74	9	16.67	54	1:1.35
May	23	41.07	27	48.21	6	10.71	56	1:1.43
June	21	43.03	25	51.22	3	5.75	49	1:1.32
July	19	37.25	30	58.82	2	3.92	51	1:1.68
August	20	44.50	22	48.95	3	6.54	45	1:1.25
September	28	45.90	31	50.82	2	3.28	61	1:1.18
October	21	51.01	17	41.30	3	7.69	41	1:0.96
November	23	32.86	42	60.00	5	7.14	70	1:2.04
December	17	34.00	25	50.00	8	16.00	50	1:1.94
Total	273		306		76		655	1:1.43

Abbreviations: M, male; NOF, non‐ovigerous female; OF, ovigerous female.

**FIGURE 3:**
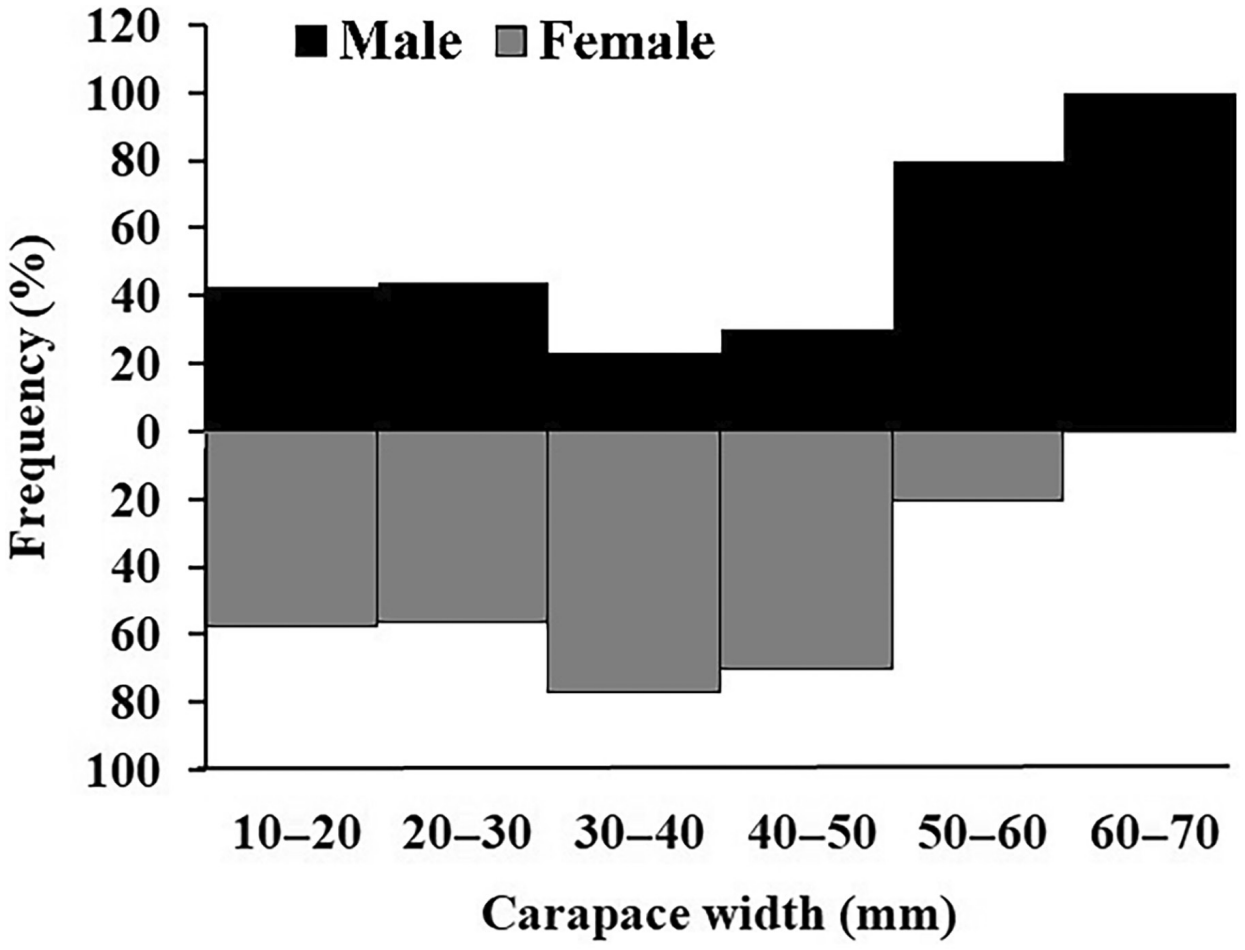
Overall size frequency distribution of *Etisus laevimanus* specimens collected from Shivrajpur, Saurashtra coast, Gujarat, India.

The sampled individuals of *E. laevimanus* were recorded from all the size classes of carapace width ranging from 10 to 70 mm. The overall frequency distribution displayed a bimodal size frequency distribution, which was observed in male individuals with highest frequency of occurrence recorded in 20–30 mm and 60–70 mm size classes. In the case of females, a unimodal size frequency distribution was observed with the highest frequency of occurrence recorded in a 30–40 mm size class (Figure 3). The size‐wise comparison of the sex ratio also revealed that the sex ratio in smaller size classes was skewed toward females, while in larger size classes, it was exclusively skewed toward males (Table 2).

**TABLE 2:** Sex ratio in different size classes of *Etisus laevimanus* specimens collected from Shivrajpur, Saurashtra coast, Gujarat state, India.

Size class (CW)	M	NOF	OF	NOF + OF	M:F
10–20 mm	3	4	0	4	1:1.33
20–30 mm	35	43	0	43	1:1.23
30–40 mm	49	125	38	163	1:3.33
40–50 mm	64	120	28	148	1:2.31
50–60 mm	99	14	10	24	1:0.24
60–70 mm	23	0	0	0	1:00

Abbreviations: M, male; NOF, non‐ovigerous female; OF, ovigerous female.

**FIGURE 4:**
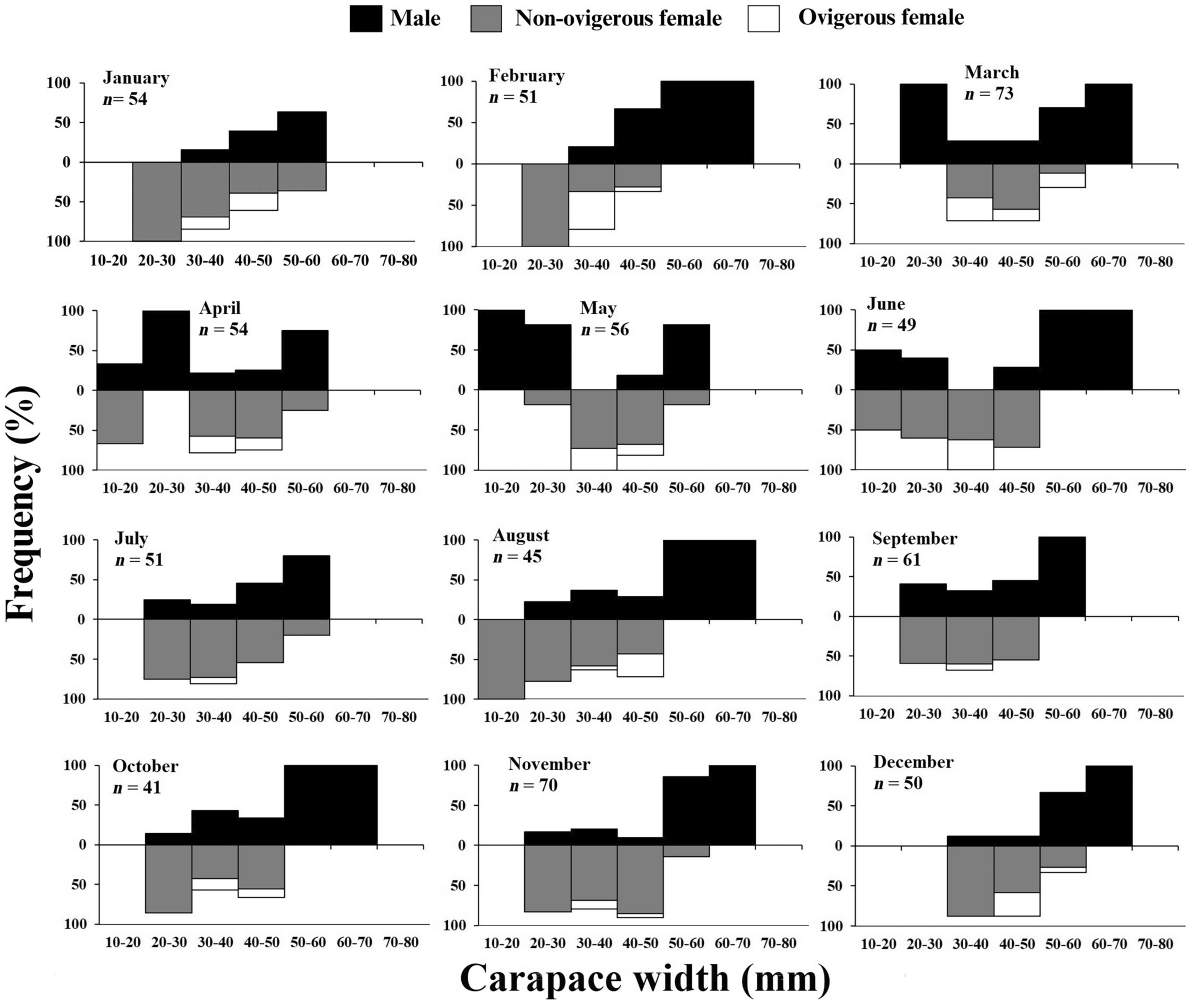
Monthly size–frequency distributions of *Etisus laevimanus* specimens collected from Shivrajpur, Saurashtra coast, Gujarat, India.

**FIGURE 5:**
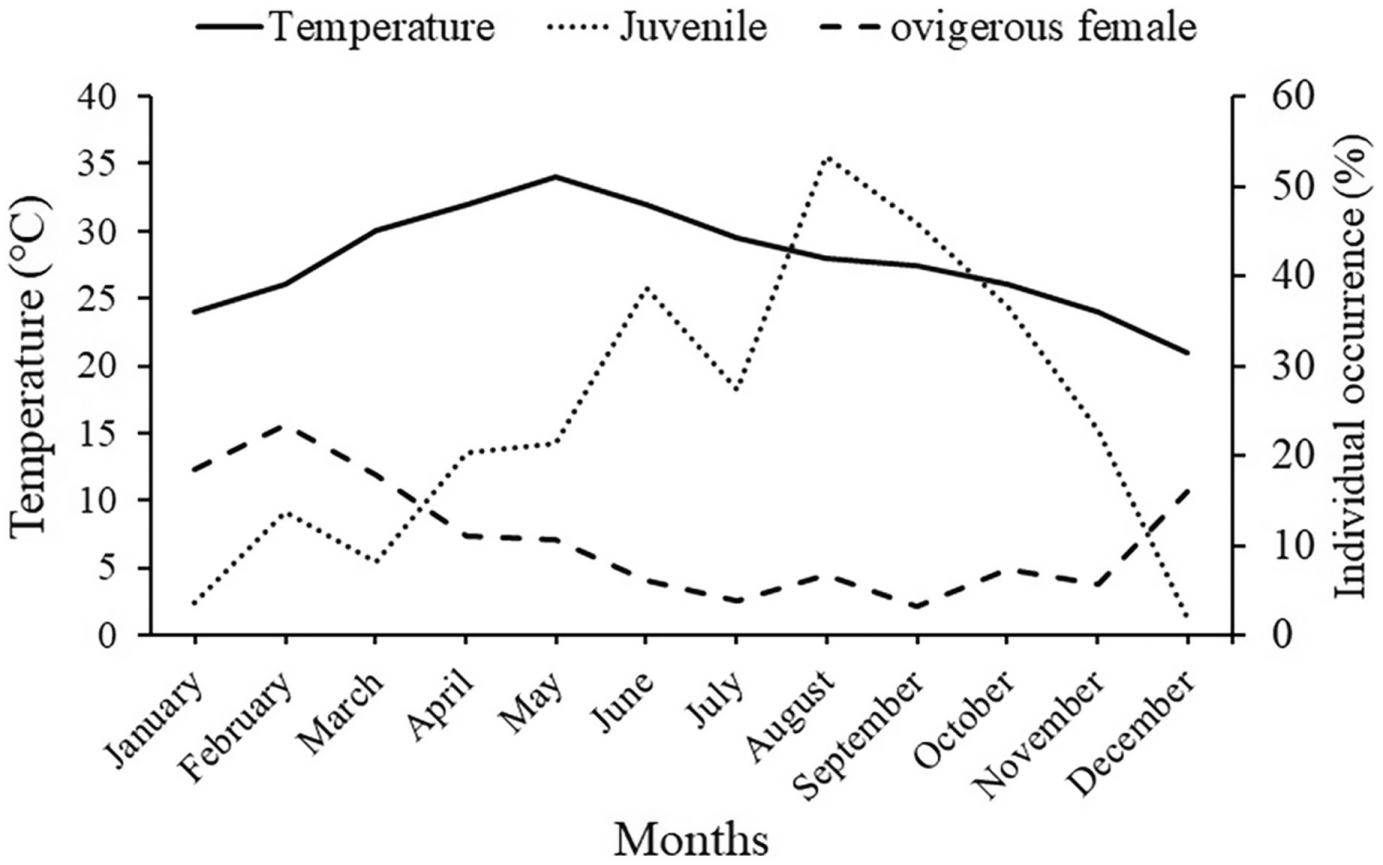
Association between the occurrence of juveniles (of both the sexes) and ovigerous females of *Etisus laevimanus* with monthly ambient temperature at Shivrajpur, Saurashtra coast, Gujarat, India.

Similarly, the monthly frequency distribution also displayed a bimodal distribution pattern in males and unimodal distribution pattern in females during the majority of the months. It was also observed that juvenile individuals (< 34.22 mm) occurred throughout the year (Figure 4). There was a significant negative relation between the occurrence of ovigerous females and juveniles (*r* = −0.78, *p* < 0.01). There was no trend observed between the monthly ambient temperature and the occurrence of ovigerous females of juveniles (Figure 5). However, it was observed that peak in juvenile recruitment was followed by the peak in the occurrence of ovigerous female. As a result of that, the percentage occurrence of juvenile individuals was less when the occurrence of ovigerous female was more (December to March) and their occurrence increased drastically with the decrease in occurrence of ovigerous females (April to November).

**FIGURE 6:**
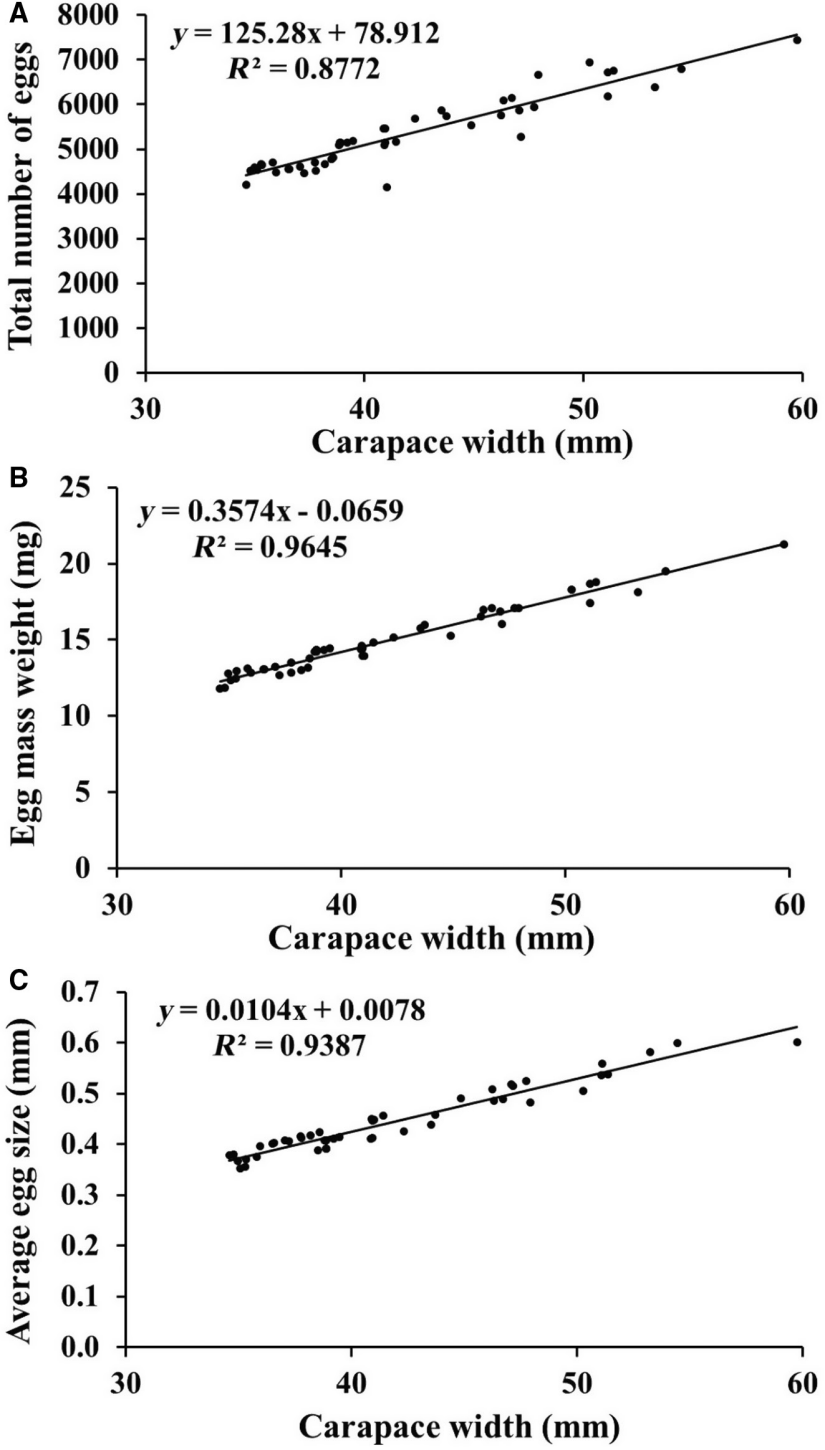
Relationship of *Etisus laevimanus* carapace width with (A) total number of eggs; (B) egg mass weight; and (C) average egg size.

The fecundity data showed that the average number of eggs on ovigerous female was 5344 ± 818.10, with the average weight of egg mass being 14.95 ± 2.23 mg and average egg size being 0.45 ± 0.07 mm (Table 3). It was found that the total number of eggs, weight of egg mass, and the size of eggs showed significantly positive relation with carapace width of the ovigerous females (Figure 6).

**TABLE 3:** Summary of ovigerous female carapace width, weight of egg mass, egg number, and egg size in *Etisus laevimanus* specimens collected from Shivrajpur, Saurashtra coast, Gujarat state, India.

	*n*	Min	Max	Mean ± SD
Carapace width (mm)	47	34.59	59.75	42.02 ± 6.11
Total number of eggs	47	4155	7438	5344 ± 818.10
Weight of egg mass (mg)	47	11.78	21.27	14.95489 ± 2.23
Egg size (mm)	47	0.35	0.60	0.45 ± 0.07

## 4 Discussion

In the current investigation, it was found that the males were significantly larger as compared to females. Similar results have been observed in several other studies conducted on crab species including *P. armatus* (Pinheiro et al. 2017), *Matuta planipes* and *Ashtoret lunaris* (Saher et al. 2017), *Scylla olivacea* (Waiho et al. 2021), *Clibanarius rhabdodactylus* (Patel, Vachhrajani, and Trivedi 2023), *Leptodius exaratus* (Patel, Patel, Ali, et al. 2024), and *Dotila blanfordi* (Patel, Patel, Gosavi, et al. 2024). Since male individuals utilize a majority of their energy for somatic growth, they can attain larger size, whereas greater energy investment of females in gonadal development leads to reduced somatic growth as compared to male individuals (Mantelatto et al. 2010). Moreover, according to sexual selection hypothesis, a larger size will aid in male–male competition for mate selection and reproductive success (Sripho and Chaiyarat 2022).

The overall as well as monthly sex ratio was significantly deviating from 1:1 and was skewed toward females, which has been observed in several other crustacean species including *Opusia indica* (Saher and Qureshi 2011) and *Macrophthalmus* (*Venitus*) *dentipes* (Qureshi and Saner 2012), *Ocypode rotundata* (Naderi et al. 2018) and *Callinectes sapidus* (Lycett et al. 2020). Moreover, the sex ratio was different in different growth stages, where smaller to intermediate size classes (10–50 mm) were female‐biased, while the larger size classes were exclusively male‐biased (50–70 mm). Female‐biased sex ratios in crustaceans including crabs can be influenced by various factors including ecological conditions and limited dispersal abilities, which leads to stronger isolation and favoring female dominance (Nijman and Vonk 2022). Numerous factors including competition in local mate (Hamilton 1967), difference in the investment in male and female offspring (Kobayashi et al. 2018), differences in the habitat utilization pattern between sexes (Silk 1984), migration patterns (Allen 1966) as well as life span, growth rates, and spatio‐temporal distribution could be one of the possible reasons for the deviation from the ideal 1:1 sex ratio (Darnell 1962; Wenner 1972; Lardies, Rojas, and Wehrtmann 1998; Wada, Kitaoka, and Goshima 2000). A female‐biased sex ratio can also be linked to survival and reproductive advantages, where female‐biased population shows increased egg production and greater survival rate, specifically in extreme habitats like the intertidal region (Ewers‐Saucedo 2019). A female‐biased sex ratio in the intermediate size class could be attributed to greater male mortality (Asakura 1992). On the other hand, a higher somatic growth rate in males leads to a male‐biased sex ratio in larger size classes (Wenner 1972). A male‐biased sex ratio in larger size classes could also be due to greater female mortality due to greater reproductive investments as well as decreased predation risk of larger male individuals possessing larger chela (Johnson 2003). The sex ratio near to the ideal 1:1 in February and October could be explained as a result of certain possible reasons. Studies suggest that environmental factors like temperature or tidal cycles can greatly affect the activity levels of crabs (Díaz and Conde 1989). Such environment factors can also affect the migration pattern, resulting in occupying similar habitats by both the sexes balancing the observed sex ratio (Hines 1982).

The current investigation showed a bimodal frequency distribution in males, but unimodal frequency distribution in females. Similar results have been observed in several other crab species like *Pilumnus vespertilio* (Litulo 2005), *Aegla franciscana* (Gonçalves, Castiglioni, and Bond‐Buckup 2006), *Aegla georginae* (Copatti et al. 2016), *C. rhabdodactylus* (Patel, Vachhrajani, and Trivedi 2023), *L. exaratus* (Patel, Patel, Ali, et al. 2024), and *D. blanfordi* (Patel, Patel, Gosavi, et al. 2024). Various explanations have been proposed to explain such type of distributions, including differences in migratory patterns (Flores and Negreiros‐Fransozo 1999), growth rates (Negreiros‐Fransozo, Costa, and Colpo 2003), and mortality rates (Díaz and Conde 1989). It is typically observed in organisms that reproduce multiple times in each season and produce a large number of clutches (Zimmerman and Felder 1991). Unimodality occurs in stable populations with equal numbers of immigrants and emigrants, consistent recruitment and mortality rates throughout the life cycle, and stable demographics (Thurman 1985; Díaz and Conde 1989), while bimodality indicates general population growth trends. Also, over time, the population size and frequency of dispersion may be significantly changed by the rapid recruitment of larvae and reproductive rate (Thurman 1985).

The temperature ranged between 21°C and 34°C, which is falling in the tropical‐sub tropical climatic conditions that can support a continuous reproduction. As a result of this in the current investigation, ovigerous females were observed in all the months of the year. Moreover, there was no correlation found between ambient temperature and occurrence of ovigerous females. Similar results have been recorded in various other crab species including *Opusia indica* (Saher and Qureshi 2011), *L. exaratus* (Al‐Wazzan et al. 2020; Patel, Patel, Ali, et al. 2024), and *Petrochirus diogenes* (Bertini and Fransozo 2002), in which there was no association between the ambient temperature and occurrence of ovigerous females of the continuous breeding species. Temperature is one of the major factors governing the abundance and distribution of the species in the intertidal region of a tropical or subtropical region where temperature rises very high (Allen 1966; Asakura 1987; Al‐Wazzan et al. 2020). Hence, seasonal fluctuation can be observed as a result of migration in population or mortality, leading to underestimation of the population during summer season (Patel, Patel, Ali, et al. 2024).

Furthermore, there was a significantly negative correlation observed between the frequency occurrence of ovigerous females and juveniles, suggesting that the juvenile occurrence decline when occurrence of ovigerous female increases and vice‐a‐versa. These results demonstrates that the species has continuous juvenile recruitment period, which could be the result of rapid reproduction and short incubation time. Such results have also been observed in other species including *Scylla olivacea* (Rouf et al. 2021), *D. japonicus* (Oh and Lee 2020), *C. rhabdodactylus* (Patel, Vachhrajani, and Trivedi 2023), *L. exaratus* (Patel, Patel, Ali, et al. 2024), and *D. blanfordi* (Patel, Patel, Gosavi, et al. 2024).

The reproductive maxima among populations can be influenced by several factors, including salinity (Huang et al. 2022), nutritional quality of females (Matias et al. 2016), nutrition availability and quality (Viña‐Trillos, Brante, and Urzúa 2023), water temperature (Chou, Head, and Backwell 2019), photoperiod (Zhang et al. 2023), and predation rate (Touchon, Gomez‐Mestre, and Warkentin 2006). Periodicity in reproduction could be due to various biotic and abiotic factors like larval ecology (Reese 1968), food availability (Goodbody 1965), time to attain sexual maturity, mating period, gonadal development, incubation period (Sastry, Vernberg, and Vernberg 1983), and so forth.

The total number of eggs, egg mass weight, and the size of eggs showed a significantly positive relation with the CW of ovigerous females. Such results have also been reported in several previous studies (Pinheiro et al. 2017; Hamasaki, Ishii, and Dan 2021; Aviz et al. 2022; Mustaquim, Khatoon, and Rashid 2022; Patel, Vachhrajani, and Trivedi 2023; Patel, Patel, Ali, et al. 2024; Patel, Patel, Gosavi, et al. 2024). A difference in the total number of eggs and egg mass weight was observed among the ovigerous females with the same CW, possibly due to differences in food availability, disparity in egg production, or loss of eggs (Hines 1982). Since brachyuran crab fertility is influenced by various external and internal factors, it might differ across intraspecific ovigerous female individuals in the same habitat or different parts of the same habitat. Variations in fertility may result from intrinsic variables like age of sexual development, food availability, differences in overall female size, and so forth (Zairion et al. 2015). As for the external factors, they include competition within and across. Fecundity may be impacted by the energy trade‐off between somatic development and egg production (Zairion et al. 2015). Moreover, females with higher CW also lay more eggs, indicating that CW is a major contributor to fecundity variability (Muiño 2002).

## 5 Conclusion

The present research aimed at investigating the population structure and breeding biology of *E. laevimanus* . Significant sexual dimorphism was observed, with males being significantly larger than females. This difference is likely due to males allocating their energy toward somatic growth, whereas females must invest their energy in egg production. The overall and monthly populations were found to be biased toward females (1:1.43). This bias can result from variations in biology and behavior, along with the influence of abiotic and biotic factors on both males and females. The presence of ovigerous females throughout the year indicates that the population is continuously breeding, and this trend appears to have an inverse relationship with the peak in juvenile recruitment, a pattern frequently seen in other tropical brachyuran crabs. There was a positive correlation between the egg mass weight, the number of eggs, and the size of the eggs with the morphology of ovigerous females. Several intrinsic and extrinsic factors, such as energy expenditure related to somatic growth and egg production, may be influencing fecundity.

## Author Contributions

**Bhavesh R. Shrimali:** conceptualization (equal), data curation (equal), formal analysis (equal), methodology (equal), visualization (equal), writing – original draft (equal). **Krupal J. Patel:** conceptualization (equal), data curation (equal), formal analysis (equal), methodology (equal), visualization (equal), writing – original draft (equal). **Ashish Patel:** data curation (equal), formal analysis (equal), resources (equal), validation (equal), writing – review and editing (equal). **Dipak Kumar Sahoo:** data curation (equal), formal analysis (equal), methodology (equal), resources (equal), validation (equal), writing – review and editing (equal). **Mansour Shrahili:** formal analysis (equal), software (equal). **Mohammad Javed Ansari:** data curation (equal), writing – review and editing (equal). **Jigneshkumar N. Trivedi:** conceptualization (equal), formal analysis (equal), methodology (equal), supervision (equal), writing – review and editing (equal).

## Conflicts of Interest

The authors declare no conflicts of interest.

## Supporting information


Data S1.

## Data Availability

The data that support the findings of this study are available from the corresponding author upon reasonable request. Specimens are deposited in Zoological Reference Collection (LFSc.ZRC), Department of Life Sciences, Hemchandracharya North Gujarat University, Patan, Gujarat, India. All the data that support the findings of this study are available in the Supporting Information.
